# Preclinical evaluation of potential therapeutic targets in dedifferentiated liposarcoma

**DOI:** 10.18632/oncotarget.10518

**Published:** 2016-07-09

**Authors:** Robert Hanes, Iwona Grad, Susanne Lorenz, Eva W. Stratford, Else Munthe, Chilamakuri Chandra Sekhar Reddy, Leonardo A. Meza-Zepeda, Ola Myklebost

**Affiliations:** ^1^ Department of Tumor Biology, Institute of Cancer Research, The Norwegian Radium Hospital, Oslo University Hospital, Oslo, Norway; ^2^ Norwegian Cancer Genomics Consortium, Oslo, Norway; ^3^ Genomics Core Facility, Department of Core Facilities, Institute of Cancer Research, the Norwegian Radium Hospital, Oslo University Hospital, Oslo, Norway

**Keywords:** FRS2, NVP-BGJ398, liposarcoma, personalized genomics, targeted therapy

## Abstract

Sarcomas are rare cancers with limited treatment options. Patients are generally treated by chemotherapy and/or radiotherapy in combination with surgery, and would benefit from new personalized approaches. In this study we demonstrate the potential of combining personal genomic characterization of patient tumors to identify targetable mutations with *in vitro* testing of specific drugs in patient-derived cell lines. We have analyzed three metastases from a patient with high-grade metastatic dedifferentiated liposarcoma (DDLPS) by exome and transcriptome sequencing as well as DNA copy number analysis. Genomic aberrations of several potentially targetable genes, including amplification of *KITLG* and *FRS2*, in addition to amplification of *CDK4* and *MDM2*, characteristic of this disease, were identified. We evaluated the efficacy of drugs targeting these aberrations or the corresponding signaling pathways in a cell line derived from the patient. Interestingly, the pan-FGFR inhibitor NVP-BGJ398, which targets FGFR upstream of FRS2, strongly inhibited cell proliferation *in vitro* and induced an accumulation of cells into the G0 phase of the cell cycle. This study indicates that FGFR inhibitors have therapeutic potential in the treatment of DDLPS with amplified *FRS2*.

## INTRODUCTION

Sarcoma accounts for approximately 1% of all cancers. However, rare cancers together make up one of the largest patient groups and are by far the largest group with regards to lost years of life. This is largely due to the many heterogeneous subgroups, making research difficult and the interest from industry low. However, deeper understanding of the mechanisms driving these cancers may reveal therapeutic targets already identified in other cancers, and thus offer new treatment options. The present work is a demonstration of the potential of tumor sequencing to identify new therapeutic targets in sarcoma patients.

Liposarcomas are the most common class of soft tissue sarcoma, accounting for about 20% of all sarcomas [1], and can be subclassified into three main histological groups: 1) well- and dedifferentiated liposarcomas (WD/DDLPS), 2) myxoid and round cell liposarcomas, and 3) pleomorphic liposarcomas [2]. WD/DDLPS is the most frequent liposarcoma subtype comprising 64% of all liposarcomas [3], and although DDLPS frequently arise from WDLPS, in many cases no WDLPS component is observed, suggesting the transformation may happen at a very early stage. Tumors in the extremities are in most cases successfully removed by surgery, whereas recurrence and progression is common for retroperitoneal tumors. Chemotherapy is usually ineffective, and progressive or metastatic retroperitoneal tumors are in most cases fatal. New therapeutic treatments are therefore desperately needed.

The molecular and cytogenetic profiles of WDLPS- and DDLPS are similar, a relatively normal karyotype, but with giant marker neochromosomes containing multiple amplified segments from various chromosomes, including universal amplification of multiple segments in the 12q13-q15 region [4–8]. This region contains known proto-oncogenes like *MDM2*, *CDK4* and *HMGA2* [4, 9–14]. The main difference between WDLPS and DDLPS is that WDLPS consists of locally aggressive but quite mature adipose-like tissue, whereas DDLPS is a high-grade undifferentiated tumor with metastatic potential [15].

In this study we investigated the case of a patient with high-grade metastatic retroperitoneal DDLPS with previously confirmed amplification of *MDM2* and *CDK4*. The patient was unsuccessfully treated with a Nutlin-3 derivative (RG7112) and Palbociclib, inhibitors of these targets, respectively. We have also investigated the possible causes for therapy failure and identified other potential therapeutic targets, through experimental investigation *in vitro* using a patient-derived cell line.

## RESULTS

### Disease history and biological material

The patient (Female, 57 years of age) diagnosed with high-grade metastatic DDLPS in the left peritoneum had previously undergone surgery and been treated with a variety of chemotherapeutic agents including Doxorubicin (Doxil), Trabectedin (Yondelis) and Ifosfamide (Ifex) without showing any improvement. During subsequent surgery the left portion of the diaphragm and 20% of the stomach were removed, followed by the treatment with gemcitabine (Gemzar), Docetaxel (Taxotere), DTIC (Dacarbazine) and the CDK4 inhibitor Palbociclib. The patient subsequently received the MDM2 inhibitor RG7112 (RO5045337), a Nutlin-3 derivative, together with Doxorubicin, also with no response.

After treatment with RG7112 and Palbociclib the disease progressed rapidly and the patient underwent surgery at which the tissue from three of the metastases was obtained for this study. The metastatic tumors, which were all classified as DDLPS, were situated at the ileocecal valve (B), the left peritoneum (D), and diaphragm (K). A patient-derived xenograft model was generated from tumor B in nude mice and a cell line named NRH-LS1 was derived from the xenograft. Tumors B, D and K were furthermore analyzed by exome and transcriptome sequencing as well as DNA copy number analysis.

### Detection of somatic single nucleotide variations

To identify somatic mutations, whole exome sequencing was performed on DNA from normal blood and metastatic tumors B, D and K with 50, 43, 42 and 35 million reads, resulting into a mean coverage of 50, 43, 44 and 34×, respectively. More than 90% of the reads could be uniquely aligned to the genome. In tumor B we detected 428 somatic single nucleotide changes (SNVs), in tumor D 391 and tumor K 385 SNVs, in total identifying 1014 unique changes (Supplementary Table 1). Somatic changes were annotated using the Oncotator web application [16]. Most SNVs were located within introns, followed by 3′ UTRs and coding regions. Among the mutations within coding regions, the majority of SNVs were missense mutations (Supplementary Table 1). Only 58 of the SNVs were shared by all three tumors, and approximately 200 were shared by at least two tumors (Supplementary Table 1). Among the mutations common for all the 3 tumors, 22 were located in protein coding regions or splice sites (18 missense, 3 silent and 1 splice site, Supplementary Table 2), and only one of these variants was present in the COSMIC database, corresponding to maltase-glucoamylase (*MGAM* p.R384H).

The mRNA expression of the coding SNV alleles was analyzed using RNA-seq data from tumor B. Of the 93 SNVs within coding regions or splice sites, 22 were expressed, 8 of which were shared by all tumors. Six corresponded to missense mutations, located in the genes *DOCK7*, *TSN*, *SVIL*, *NDUFA9*, *TPP2*, *COG1*, a silent mutation in *DDX11* and a splice site mutation in *EDEM1* that resulted in exon skipping (Supplementary Table 2). All somatic mutations expressed in tumor B were also expressed in the derived cell line NRH-LS1, whereas *MGAM* was neither expressed in tumor B nor in the cell line.

### DNA copy number and RNA-seq analysis

**Figure 1:**
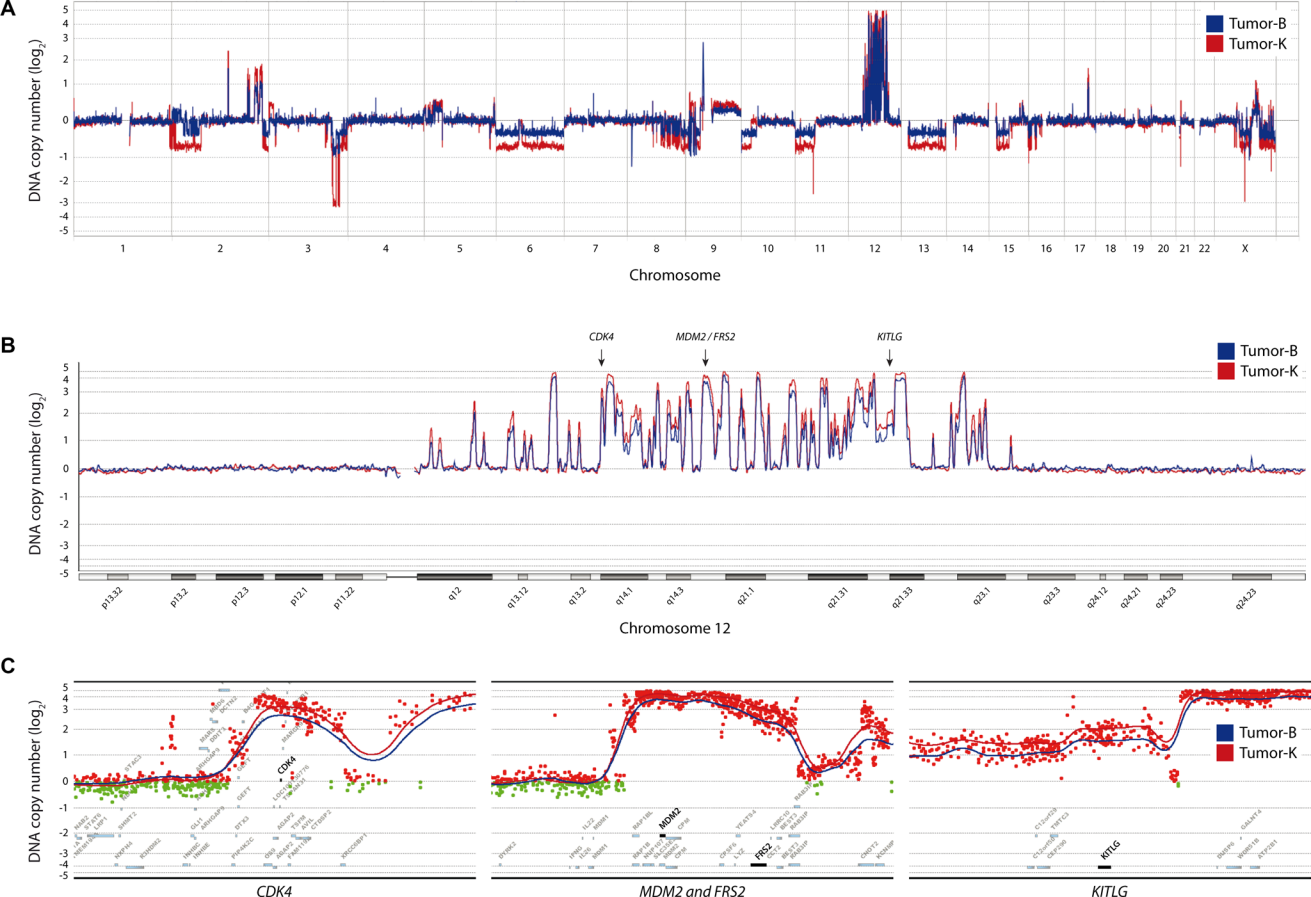
DNA copy number changes for tumor-B (blue) and -K (red) (**A**) Genome-wide DNA copy number plot. (**B**) Landscape view of chromosome 12 with high-level amplification for *CDK4*, *MDM2*, *FRS2* and *KITLG*. (**C**) Detailed view on the flanking genomic region for all four genes; red dots indicate data points from either tumor with a log_2_ ratio > 0 and green dots with log_2_ ratio < 0. The ratio is plotted according to position with triangular smoothing of 200 kb, with blue line indicating tumor B, red tumor K.

DNA copy number changes for samples B and K were mapped at high resolution using comparative genomic hybridization (CGH) microarrays (Figure 1). There were more genomic losses than gains, with copy number aberrations on almost every chromosome, but with the typical large number of high-level amplifications (log_2_ ratio > 0.8) on chromosome 12 characteristic for WD/DDLPS (Figure 1B). Multiple focal high-level amplifications were also seen in 2q, 17q and Xq in both samples. Large regions of loss were detected in 2p, 3q, 6, 8q, 9p, 10p, 11p, 13, 15q and Xq, with small homozygous regions (log_2_ ratio < −0.8) in 3q26.1, 3q26.31, 3q26.32, 9p23, 9p22.3, 9p21.3 and 9p21.2. Overall, the similarity in DNA copy number changes between sample B and K was high, sample K presenting increased amplitude in all amplified and deleted regions compared to sample B, most likely due to differences in tumor cell content. Specific changes were observed in 2p, where sample K showed a large heterozygous deletion in 2p12-p24.3, while sample B presented smaller focal deletions within this region. Additional minor differences in copy number between sample B and K were observed across various chromosomes (Supplementary Table 3).

Analysis of regions of high-level amplification and homozygous deletion identified 230 genes with increased copy number and 61 with loss. Gene expression analysis by RNA-seq showed increased expression in almost every amplified gene when compared to an undifferentiated immortalized mesenchymal progenitor cell line (iMSC#3, [17, 18]) (Supplementary Table 4), which resembles the expected cell of origin for this tumor type. Conversely, a clear reduction of expression compared to iMSC#3 was seen for approximately half of the genes within homozygously deleted regions. The residual expression of the remaining genes is most likely from stroma cells within the tumor. RNA-seq also identified a number of expressed fusion transcripts, involving genes mostly in 12q12-22 (data not shown).

Among the amplified and overexpressed genes were *MDM2*, *CDK4* and *HMGA2*, all well-known amplified targets in liposarcoma. Further analysis identified additional amplified and overexpressed genes in potentially targetable pathways. One of those genes was *FRS2,* localized within the same 12q15 amplicon as *MDM2* and coding for fibroblast growth factor receptor substrate 2, an adapter protein that links the fibroblast growth factor receptor (FGFR) to downstream signaling pathways. Another gene was *KITLG*, localized in 12q22, coding for the ligand of the tyrosine-kinase receptor c-KIT, and *STAT1* and *STAT4*, both members of the signal transducers and activators of transcription family, localized in 2q32. The most relevant amplified segments are shown in more detail in Figure 1C.

In addition to MDM2 and CDK4, we considered KITLG and FRS2 candidate therapeutic targets that we investigated further. Array CGH analysis of sample B showed that *KITLG* and *FRS2* were amplified with a log_2_ ratio of 1.6 and 2.2, respectively. The expression level based on RNA-seq showed that *KITLG* was overexpressed at a log_2_ ratio of 1.2 and *FRS2* at a ratio of 4.8 in NRH-LS1 cells relative to undifferentiated iMSC#3 (Supplementary Table 4), with a mean transcript levels of 1038 and 757 RPKM (sequence Reads Per Kilobase of transcript per Million mapped reads), respectively.

### The patient-derived cell line responds to Nutlin-3, but not to Palbociclib

**Figure 2:**
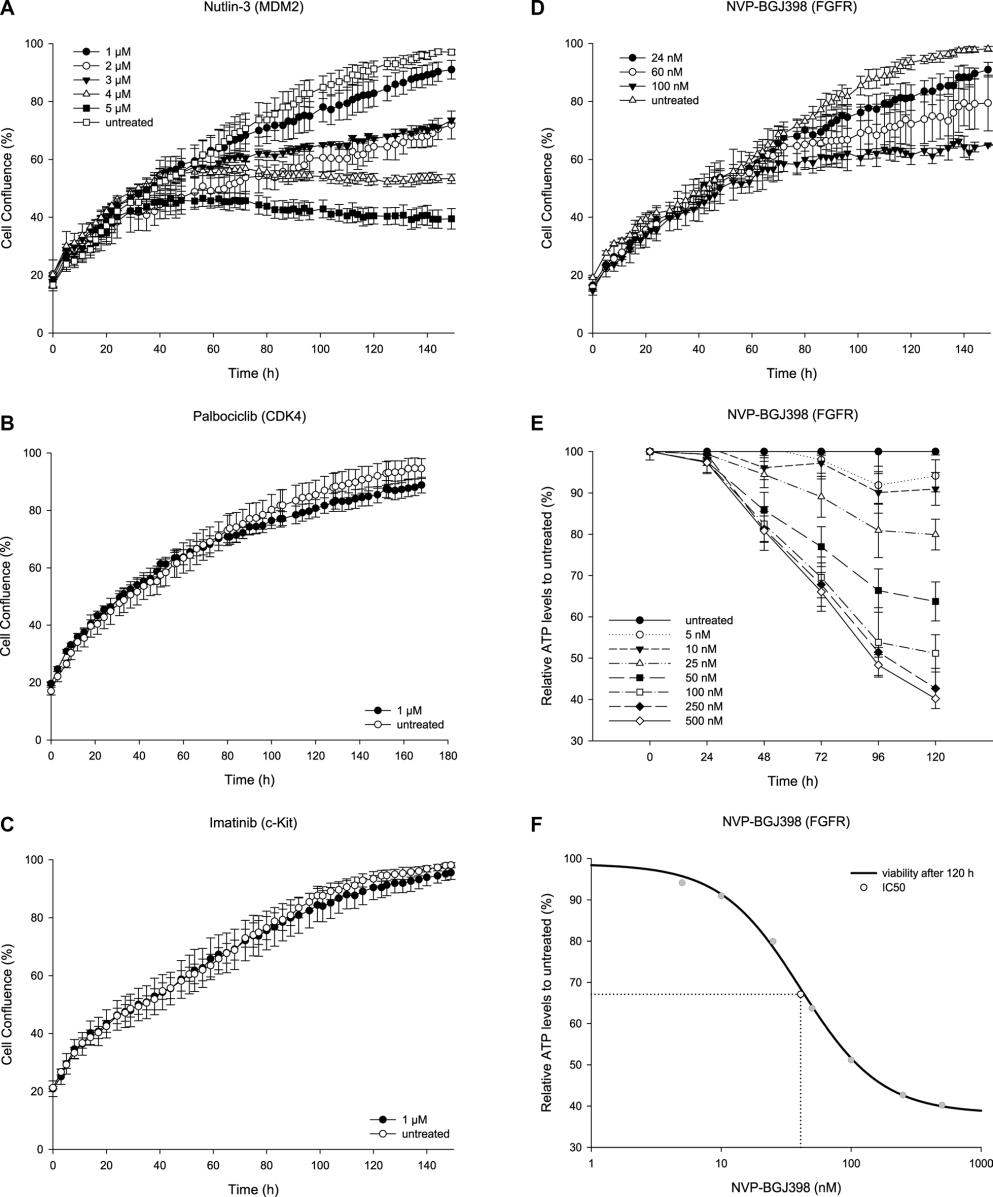
The therapeutic potential of targeted inhibition on the proliferation of NRH-LS1 cells *in vitro* (**A**) Proliferation of NRH-LS1 cells after inhibition of MDM2 with Nutlin-3; one representative experiment is shown (*n* = 4). (**B**) Inhibition of CDK4 with Palbociclib; one representative experiment is shown (*n* = 4). (**C**) Targeting KITLG through c-KIT inhibition using Imatinib; one representative experiment is shown (*n* = 4). (**D**) Proliferation of cells after FGFR inhibition with NVP-BGJ398; one representative experiment is shown (*n* = 8). (A–D) Proliferation measured based on confluence over time; error bars represent standard error (SE) of the measurements. Untreated with DMSO concentration corresponding to that of the highest drug concentration. (**E**) Viability of cells based on ATP measurement at different time points after treatment with various doses of NVP-BGJ398; (*n* = 1); error bars represent standard deviation (SD) of the measurements. (**F**) The IC_50_ was estimated at 40 nM based on cell viability after 120 h of treatment with NVP-BGJ398; (*n* = 1).

The patient had received drugs targeting MDM2 and CDK4 but with no therapeutic effect. To investigate if the lack of response was due to intrinsic drug resistance, we treated the NRH-LS1 cell line with Nutlin-3 and Palbociclib *in vitro*. The cells were sensitive to Nutlin-3 in a dose-dependent manner between 1 and 5 μM (Figure 2A), which is consistent with other WD/DDLPS cell lines [19–21]. Surprisingly, despite the high level of *CDK4* amplification and expression, the NRH-LS1 cells were insensitive to Palbociclib (concentrations ranging from 0.1 to 5 μM) (Figure 2B), possibly due to a hemizygous deletion of *RB1* (Supplementary Figure 1A).

### Targeting cells with KITLG amplification through inhibition of c-KIT using Imatinib

We hypothesized that overexpression of KIT-ligand might provide an autocrine loop, making the wild type c-KIT protein a target for inhibition. This pathway is already highly relevant in some sarcomas, since mutated c-KIT is a known driver oncogene in gastrointestinal stromal tumors (GIST) [22]. The expression of *KIT* in tumor B was similar to that found in the undifferentiated iMSC#3. The kinase inhibitor Imatinib, which is successfully used to treat GISTs with mutated c-KIT [23], did however have no effect on the cell growth of NRH-LS1 cells (Figure 2C).

### Targeting cells with FRS2 amplification through inhibition of FGFR using NVP-BGJ398

Overexpressed FRS2 would be expected to provide a pathologically sustained signal to the PI3K/Akt and MAP kinase pathways, but no drug targeting FRS2 itself is currently available. Although FRS2 is downstream of FGFR, which in this case was wild type, signaling might still depend on the kinase activity of FGFR. DNA copy number analysis showed normal numbers for *FGFR1,* FGFR2 and FGFR3, while *FGFR4* showed gain with a log_2_ ratio of 0.5. However, only *FGFR1* was expressed at high level, with a mean transcript expression of 187 RPKM in NRH-LS1 (and 143 in tumor B), compared to 0.3, 47, and 7.9 for *FGFR2, FGFR3* and *FGFR4*, respectively. Compared to iMSC#3, *FGFR1* was overexpressed at a log_2_ ratio of 2.3, while *FGFR2, FGFR3* and *FGFR4* were expressed at ratios of 0.1, 4.4, and 2.4, respectively. The pan-FGFR inhibitor NVP-BGJ398 had a profound dose-dependent effect on the proliferation of NRH-LS1 cells *in vitro*, plateauing at a concentration of 100 nM (Figure 2D and 2E) and with an IC_50_ of approximately 40 nM (Figure 2F).

### FGFR inhibition with NVP-BGJ398 induces a moderate level of apoptosis

**Figure 3:**
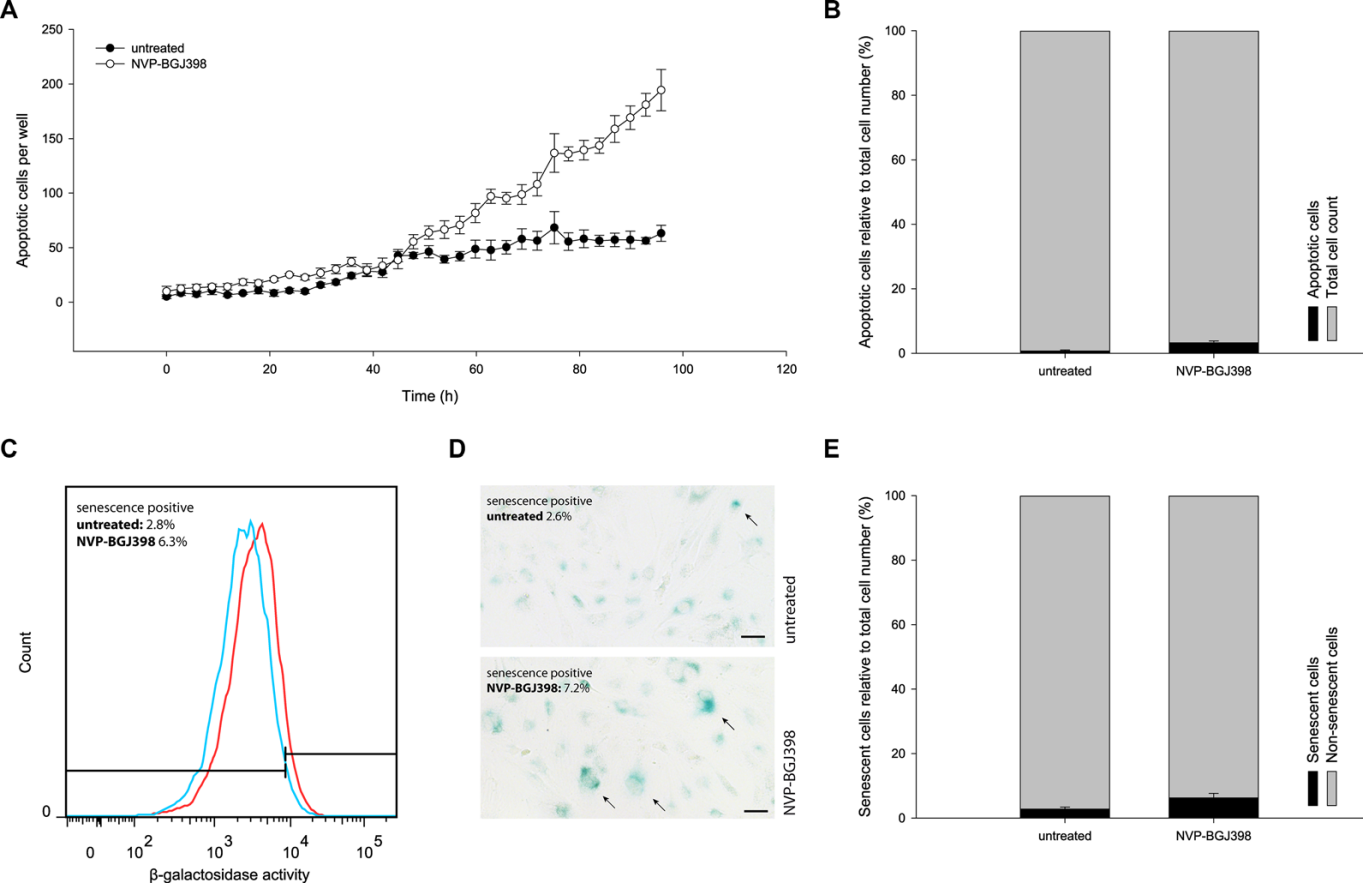
Treatment with NVP-BGJ398 does not induce NRH-LS1 cells to undergo apoptosis or senescence in a manner to account for the reduced cell number (**A**) The number of cells with active caspase 3/7 during 96 h of treatment with 100 nM of NVP-BGJ398. (**B**) The percentage of apoptotic cells after treatment with NVP-BGJ398; shown one representative experiment (*n* = 3), error bars represent the standard error (SE) of the final measurement. (**C**) Increase in SA-β-galactosidase activity between cells treated with 100 nM of NVP-BGJ398 (red) and untreated (DMSO) cells (blue). Representative flow cytometry histograms of *n* = 3 biological replicates shown. (**D**) Representative image of SA-β-galactosidase staining after 72 h of treatment with 100 nM NVP-BGJ398. Senescent cells marked with arrows. Scale bars represent 50 μm. (**E**) The percentage of senescent cells after treatment with NVP-BGJ398 based on flow cytometry assay (*n* = 3), error bars represent the standard deviation (SD) from three independent experiments.

Apoptosis was measured after treatment with 100 nM of NVP-BGJ398 based on the presence of active Caspase-3/7 using live-cell imaging (Figure 3A). Apoptosis was induced in approximately 3% of drug treated cells, compared to less than 1% of untreated cells (Figure 3B). Therefore, apoptosis is a minor factor and cannot alone account for the observed growth inhibition.

### FGFR inhibition halts cell-cycle progression and constrains cells in growth arrest during NVP-BGJ398 treatment

**Figure 4:**
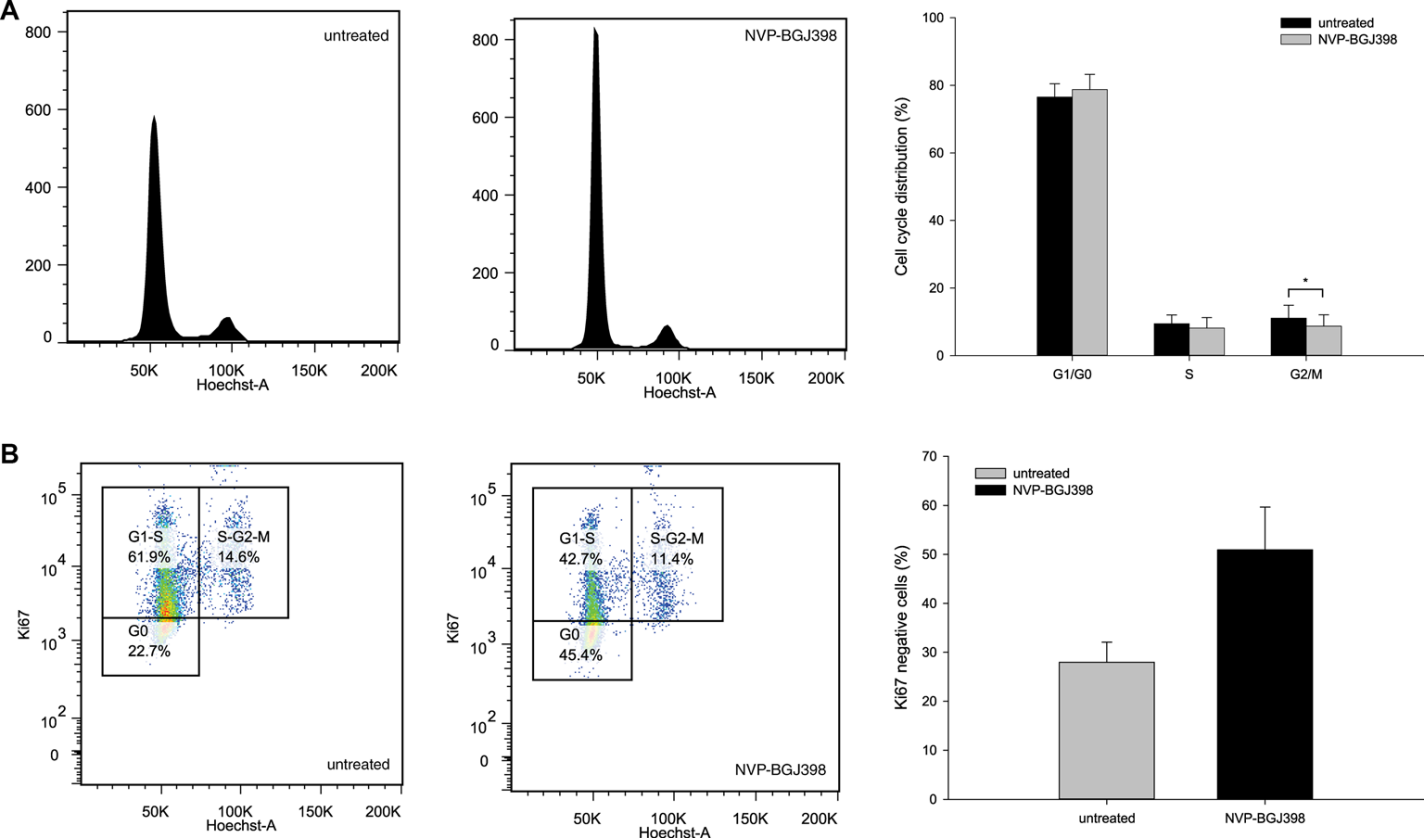
Treatment with NVP-BGJ398 affects cell cycle distribution by increasing the number of cells in G_0_ of the cell cycle (**A**) Inhibition of FGFR causes a small but significant (*p*-value < 0.05) change in G_2_/M phase distribution between cells treated with 100 nm NVP-BGJ398 and untreated (DMSO) cells. (**B**) Treatment with NVP-BGJ398 leads to a higher number of cells to accumulate in G_0_ within the G_0_/G_1_-S fraction of the cell cycle as compared to untreated (DMSO) cells. (A–B) Cells were treated with 100 nM of NVP-BGJ398 for 72 h. Representative plots (left) and bar graphs (right) of five independent experiments (*n* = 5) shown, error bars represent the standard deviation (SD) from five independent experiments. Student's *t*-Test. *p ≤ 0.05. *p*-value was determined as described in the Methods section.

Flow cytometric analysis revealed that after treatment there was a small, but significant decrease of NRH-LS1 cells in G_2_/M phase (from 11.0 to 8.7%), and a consistent but not significant increase of cells in G_1_/G_0_ after drug treatment (Figure 4A). The fractions of non-proliferating (G_0_) and proliferating, Ki67-positive (G_1_) cells was determined. Following treatment with 100 nM of NVP-BGJ398 for 72 h, approximately 45% of the cells were in G_0_ compared to 23% of the untreated cells (Figure 4B). Cells in G_0_ may either be quiescent or senescent, and therefore either temporarily or permanently arrested.

Senescence-associated β-galactosidase (SA-β-gal) activity was measured by flow cytometry in live cells after drug treatment. There was an increase of median SA-β-gal activity following treatment with 100 nM NVP-BGJ398 for 72 h (Figure 3C). However, a weak but clearly positive staining of all cells complicated precise identification of senescent cells. Therefore we also measured SA-β-gal activity using a chromogenic, microscopy-based assay (Figure 3D). The percentage of SA-β-gal positive cells was 7.2% in NVP-BGJ398-treated and 2.6% in untreated cells. Since the untreated cells showed a normal rate of proliferation and therefore should be mostly non-senescent, the overall high level of SA-β-gal activity seen with flow cytometry cannot reflect overt senescence. Therefore, we gated the flow cytometry data so that less than 3% of the untreated cells in one replicate were scored as senescence-positive (as observed by microscopy) and applied this gating to the drug-treated population. With these parameters, on average, 6.3% of the treated cells were senescent, compared to 2.8% of untreated cells (Figure 3E). Despite the doubling of senescent cells induced by drug treatment, senescence only accounts for a small fraction of the decrease in proliferation.

Therefore it appears that a large fraction of NRH-LS1 cells treated with NVP-BGJ398 are only temporarily arrested by FGFR inhibition.

### Recovery of proliferation in NRH-LS1 cells after withdrawal from FGFR inhibition

**Figure 5:**
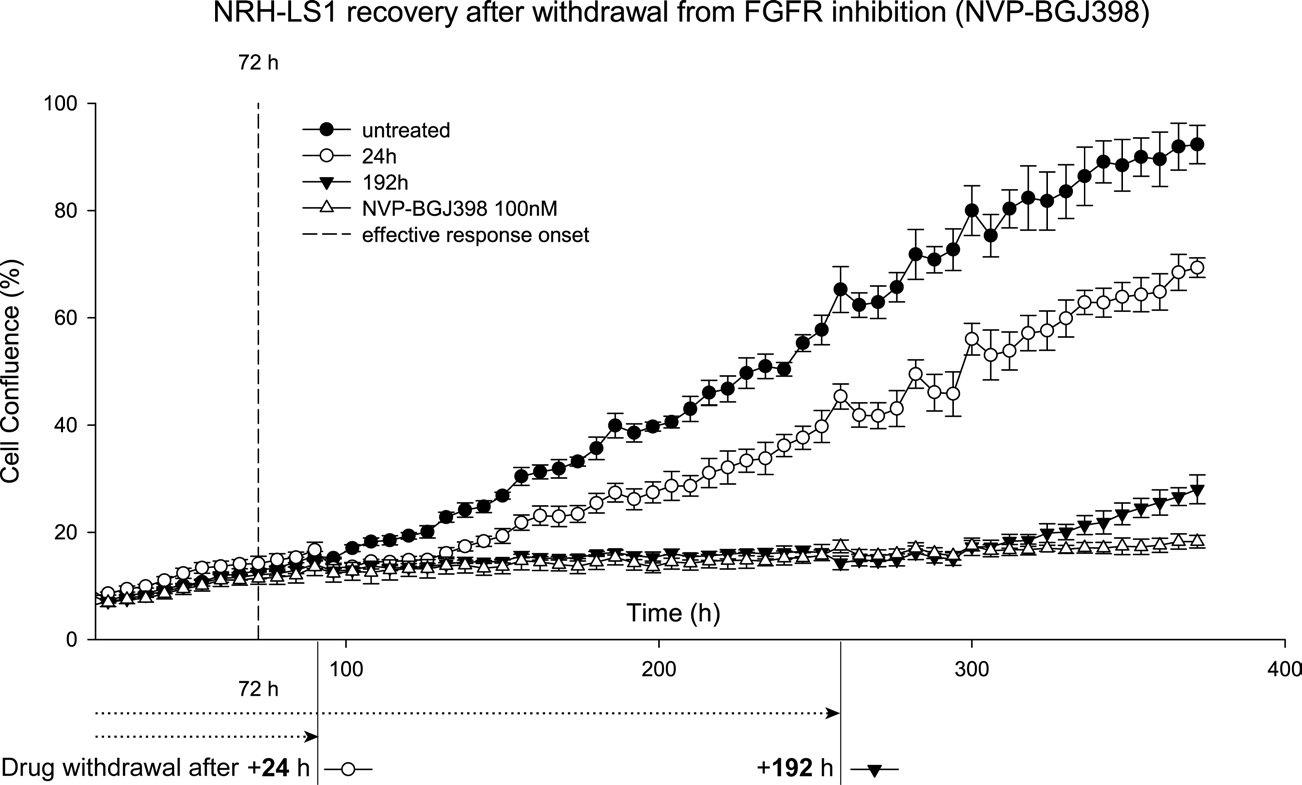
NRH-LS1 cells resume proliferation after withdrawal from treatment with NVP-BGJ398 Proliferation of NRH-LS1 cells after FGFR inhibition and withdrawal from the drug treatment after 24 h and 192 h post effective response onset (72 h); one representative experiment is shown (*n* = 2). Proliferation measured based on confluence over time; error bars represent standard error (SE) of the measurements. Untreated with DMSO concentration corresponding to that of the highest drug concentration.

To determine the reversibility of the cell cycle arrest, NRH-LS1 cells were exposed to 100 nM of NVP-BGJ398 for 96 to 264 h, followed by withdrawal of the drug. After the recovery from the drug treatment, NRH-LS1 cells regained at all time points their usual proliferative potential (Figure 5).

### Other DDLPS cell lines with FRS2 amplification show variable response to FGFR inhibition

The treatment of two other DDLPS cell lines, LPS510 and LPS853, interestingly gave different results, only LPS510 showed a similar response as NRH-LS1, while LPS853 was unaffected by the inhibition of FGFR (Supplementary Figure 2). Both cell lines have a similar copy number profile and a similar expression of *FRS2* compared to NRH-LS1 (data not shown).

## DISCUSSION

This study is an extensive genome characterization of several late metastatic DDLPS tumors from a patient who had received multiple cytotoxic and targeted therapies, with no lasting efficacy. As previously described for WD/DDLPS [4, 6, 24], the three metastases had a common and very similar core set of amplified segments on chromosome 12, including *MDM2, HMGA2*, and *CDK4*. Interestingly, we did not see loss of 11q23-24, which has been associated with greater genomic complexity [26]. In addition, we also see amplification of *CPM*, which has previously been reported and shown to be important for proliferation in DDLPS [25], and hemizygous deletion of *NF1*, a tumor suppressor gene functionally implicated in different types of LPS [25]. All three metastases show regions previously reported as progression-associated CNAs, such as loss of 3q29, 9p22 and 10p15 [26]. We could not discern any clear hierarchy between the three metastases, but we found a surprisingly high level of dissimilarities with regard to point mutations, suggesting that most of these genomic changes are “noise” and not “drivers”. However, seven commonly mutated alleles with coding consequences were expressed, which could be drivers or just reflect the common origin of the tumors.

The patient had received treatments targeting CDK4 and MDM2 with Palbociclib and a Nutlin-3 derivative, respectively. Since neither of these drugs gave significant benefit, we investigated the response to these drugs *in vitro*. The patient-derived cell line showed good response to Nutlin-3 *in vitro*, indicating no intrinsic resistance, and the lack of response in the patient might have been due to dose restrictions because of adverse effects observed in clinical trials [27]. The cell line was resistant to the inhibition of CDK4 by Palbociclib. DNA copy number analysis revealed the loss of one copy of *RB1*, but no mutation in the remaining allele could be detected. Deletion of *RB1* would not be expected to be selected for in the presence of amplified *CDK4* [28]. Unfortunately, we do not have samples collected from the early tumors, and we cannot therefore evaluate whether the *RB1* deletion was preexisting or selected for during the Palbociclib treatment. However, it seems possible that haploinsufficiency of *RB1* was sufficient to make the cells insensitive to CDK4 inhibition [28]. Interestingly, in addition to the amplification of *CDK4* and the hemizygous deletion of *RB1*, we also found *CDKN2A* and *CDKN2B* deleted (Supplementary Figure 1). These genes code for p14^ARF^ [29] and p16^INK4A^, and for p15^INK4B^, respectively. Both p16^INK4A^ and p15^INK4B^ are inhibitors of CDK4 [30, 31]. The deletion of *CDKN2A* and loss of p16^INK4A^ protein was shown to predict sensitivity of melanoma cell lines to Palbociclib, while loss of *RB1* led to drug resistance [32].

The exome analysis did not reveal any good candidate therapeutic targets based on point mutations. However, since many subtypes of sarcomas, and particularly WD/DDLPS, are largely thought to be copy number-driven, we also searched for amplified targets. Since mutated KIT receptor is known to drive GIST sarcomas, it seemed possible that the moderate amplification of *KITLG* and resulting overexpression of the c-KIT ligand could be a driver, providing an autocrine loop. However, NRH-LS1 cells did not respond to the kinase inhibitor Imatinib, although this drug can efficiently treat GIST with activated c-KIT.

Among the genes amplified on chromosome 12q15 was *FRS2*, coding for the fibroblast growth factor receptor substrate 2, an adaptor protein connecting FGF receptors and their down-stream signaling cascades [33]. This aberration has previously been shown to be a driver in ovarian cancer [34] and the amplification is frequently observed in WD/DDLPS [35]. Previous studies have shown that FRS2 and FGFR are highly expressed and active in several high-grade liposarcomas, and targetable by the FGFR inhibitor NVP-BGJ398 in DDLPS cell lines *in vitro* [36].

We show that the pan-FGFR inhibitor NVP-BGJ398, a small molecule kinase inhibitor targeting the ATP-binding domain of FGF receptors currently in clinical phase II, showed promising inhibition of proliferation of our patient-derived cell line *in vitro*. Further investigation revealed that the main factor contributing to the reduced proliferation was not apoptosis or senescence, but a reversible cell cycle arrest in the G_1_ and G_0_ phases. It is surprising that in spite of the multitude of amplicons, the cells appear to be dependent on FGFR signaling for proliferation. At this point it is not clear that the drug acts exclusively on FGFR, or that the signaling is dependent on the amplified and overexpressed FRS2, although a previous study substantiates such an interpretation [36].

The consequences of a G_0_/G_1_ arrest *in vivo* is yet unknown, and a reversible inhibition may stabilize disease, although induction of apoptosis would be better for clinical efficacy. Therefore combination therapy, such as with a more tolerable dose of Nutlin-3 might improve outcome by also inducing apoptosis. Furthermore, both the level of FGF signaling and the actual response to its blockade need to be determined *in vivo*. Having established xenograft models from this patient tumor as well as from other DDLPS cases [37], we will investigate the effect of FGFR inhibition *in vivo* in future studies.

FGF receptors are mutated and amplified in various cancer types [38]. *FGFR1* was shown to be amplified in breast cancer [39] and lung cancer [40, 41], *FGFR2* in breast cancer [42] and gastric cancer [43, 44]. However, to our knowledge, none of the *FGFR*s are usually amplified in liposarcoma. We also showed that this FGFR inhibitor does not arrest all *FRS2* amplified DDLPS cell lines, and that additional biomarkers may therefore be needed for clinical use. However, the inhibition of the FGFR pathway may open new opportunities for treatment of this patient group, which is currently lacking good therapeutic options. Firstly we would need to demonstrate efficacy in PDX models *in vivo*, and then we suggest an international consortium, e.g. the World Sarcoma Network^1^ to take on small-scale trials of this and similar targeted therapies on patients with the appropriate biomarkers. Regrettably, our studies took too long to be of any benefit to the patient, who succumbed to the disease during our work.

## MATERIALS AND METHODS

### Patient samples

Patient samples and clinical information were obtained upon a written consent and according to approval no. S-06133 from the Regional Ethics Committee for Medicine in Southeastern Norway. During surgery, in total 12 tumor samples were resected and labeled alphabetically from A – L. We received specimens from sample B, a tumor (1.7 cm in size) from a nodule at the ileocecal valve, sample D, a recurrent tumor (19.8 cm in size) from the left perineum, and sample K, a tumor (12.0 cm in size) from a diaphragmatic left thorax mass. All received tumor samples were classified as high-grade metastatic dedifferentiated liposarcoma.

### Exome sequencing

High quality genomic DNA was isolated using the Promega Wizard Genomic DNA Purification Kit (Promega, Wisconsin, United States) and the QIAamp DNA FFPE Tissue kit (Qiagen, Venlo, Netherlands) as previously described [47]. One microgram of genomic DNA was used to produce exome captured sequencing libraries using the Agilent SureSelect Human All Exon v5 kit (Agilent Technologies, California, United States). Paired-end 100-bp sequencing of each exome capture library was done using an Illumina HiSeq 2500 instrument and Illumina's TruSeq SBS v3 chemistry (Illumina, California, United States).

### RNA sequencing

RNA sequencing libraries were generated from 1 μg total RNA of tumor B, which was the only tumor sample providing sufficient RNA quality, and the patient-derived cell line NRH-LS1, using the Illumina TruSeq RNA Sample Preparation Kit v2 (Illumina, California, USA). The libraries were sequenced on a HiSeq 2500 Illumina sequencer using TruSeq SBS v3 chemistry generating paired-end 2 × 100 bp sequences. RNA-seq reads were aligned by Bowtie 2 [45] against the human Ensembl transcriptome version 69, and normalized RNA-seq counts were determined by DESeq [46].

### Array CGH analysis

The Agilent SurePrint G3 Human CGH 2 × 400 k microarray (Agilent Technologies, California, United States), containing 411,000 *in situ* synthesized 60-mer oligonucleotides spanning the genome, was used to determine DNA copy number changes. 500 ng of high quality genomic DNA was used to compare tumors to leukocyte DNA from the patient. Aberrations were detected using the Agilent Genomics Workbench v7.0.4.0. AMD-2 algorithm with a threshold of 11.2, and a filter against aberrations with less than six probes was used.

### Alignment, and SNV/Indel calling

Reads from tumor and matched normal blood sample were aligned separately to the human NCBI Build GRCh37 reference genome using Novoalign (Novocraft Technologies, Selangor, Malaysia) with default parameters. PCR duplicates, improper pairs and ambiguously mapped reads were removed using in-house scripts. SNVs were called using MuTect [48]. Variants annotation was done using Oncotator [16].

### Xenografts

Tissue pieces from each metastatic sample were implanted subcutaneously on the flank of locally bred athymic nude Foxn1^nu^ mice to establish patient derived xenograft models. 6 months post implantation we observed growth for tumor B, whereas the other samples did not engraft within ten months. All procedures involving animals were approved by the Institutional Animal Care and Use Committee (IACUC) and performed according to protocols approved by the local animal care unit at the Norwegian Radium Hospital, Oslo University Hospital, according to the National Ethics Committee's guideline on Animal Welfare (FDU approval number: 3275).

### Cell line establishment and culture conditions

To conduct *in vitro* analyses, we generated a cell line (NRH-LS1) from the patient-derived xenograft of tumor B. Cells were extracted from the xenograft as previously described [49]. To ensure the cell line was not containing cells of murine origin, the cells were stained with anti-TRA-1-85 (Beckton Dickinson, New Jersey, USA) and analyzed by flow cytometry [49]. Cell lines were cultured in RPMI-1640 medium, with sodium bicarbonate and without L-glutamine (Sigma-Aldrich, St. Louis, USA) supplemented with 10% FBS (Sigma-Aldrich, St. Louis, USA), 1% L-Alanyl-L-Glutamine (Sigma-Aldrich, St. Louis, USA) and 1% Penicillin-Streptomycin (Sigma-Aldrich, St. Louis, USA). Cells were grown at 37°C, 5% CO_2_. All experiments using NRH-LS1 have been conducted on cells between passage 10 and 30. Different cell passages have been compared without any difference in outcome for individual experiments. Short tandem repeat (STR) DNA profiling confirmed that the STR-DNA profile between the cell line, the tumor sample and normal blood was the same. Cells were confirmed negative for mycoplasma using the VenorGeM Mycoplasma Detection Kit (Minerva Biolabs, Berlin, Germany).

### Drugs

Palbociclib (PD-0332991)(#S1116), Imatinib (STI571)(#S2475), Nutlin-3 (#S1061), and NVP-BGJ398 (#S2183), were all purchased from Selleck Chemicals (Munich, Germany) and dissolved in DMSO (Sigma-Aldrich, Missouri, USA) according to the manufacturers recommendation.

### Drug treatment and cell proliferation assay

The cellular proliferation rate was measured using a live-cell imaging system, IncuCyte ZOOM (Essen Bioscience, Birmingham, UK) with the corresponding software application (version 2013BRev1). For each experiment an equal number of cells were seeded (5 × 10^3^ cells per well) onto a 96-well plate. The drug treatment was initiated between 16 and 20 h after seeding and applied at different concentrations. For each drug concentration medium with the appropriate DMSO concentration was used as a control. The proliferation rate was measured as cell confluence over time through the acquisition of photographs under phase contrast every third hour for the entire duration of the drug treatment.

### Apoptosis

The apoptosis assay was performed using a live-cell imaging system, IncuCyte ZOOM (Essen Bioscience, Birmingham, UK). For each experiment an equal number of cells have been seeded (4 × 10^3^ cells per well) onto a 96-well plate. The drug treatment was carried out as previously described with either 100 nM of NVP-BGJ398 or 0.01% DMSO. Apoptosis was measured using the CellPlayer 96-Well Kinetic Caspase-3/7 reagent (Essen Bioscience, Birmingham, UK), containing DEVD-NucView^TM^488 in DMSO. The total number of apoptotic cells was counted on the green channel at 488 nm. The total cell count was measured on the red channel at 566 nm after 96 h of drug treatment by incubating cells for 30 minutes with 4 μM of Nuclear-ID Red DNA stain (Enzo Life Sciences, NY, USA). The percentage of apoptotic cells per well was calculated as the number of apoptotic cells relative to the total number of nuclei.

### Senescence assay

Cellular senescence was measured with the Cellular Senescence Live Cell Analysis Assay Kit (Enzo Life Sciences, NY, USA), using a fluorogenic β-galactosidase substrate. 8 × 10^4^ (drug treated) and 6 × 10^4^ (untreated) cells were seeded onto a 6-well plate and the drug treatment was carried out as previously described with either 100 nM of NVP-BGJ398 or 0.01% DMSO. 96 h post drug treatment, cells were incubated for 2 h in pretreatment solution. The fluorogenic substrate was added to the cells for additional 4 h. 1 × 10^5^ cells per sample were analyzed using LSR II Flow Cytometer (BD Biosciences, NJ, USA) flow cytometer with laser 488 and emission filter 525/50 nm. The data were processed by FlowJo Version 7.6.5 (Tree Star, Ashland, USA). Results of three independent experiments with five replicates in total are presented as a median fluorescence change ± standard deviation (SD) after the drug treatment. Calculation of the percentage of senescent cells was performed based on gating that gave 97% of senescence-negative untreated cells. Statistical analysis was performed using two-tailed, paired Student's *t*-Test. *p*-values < 0.05 were considered significant.

For the chromogenic assay of senescence associated β-galactosidase activity, the Senescence Detection Kit (BioVision, California, USA) was used. 4 × 10^4^ (drug treated) and 3 × 10^4^ (untreated) cells were seeded onto a 12-well plate and the drug treatment was carried out as previously described with either 100 nM of NVP-BGJ398 or 0.01% DMSO for 72 h. Pictures from nine random fields of view were taken using an Olympus IX-81 inverted fluorescence microscope with the Olympus DP72 camera (Olympus, Tokyo, Japan) and the Olympus cell^P^software (version 3.4) (Olympus Soft Imaging Solutions, Münster, Germany) at a magnification of 10× and scored by counting senescence positively stained cells. The scoring was done blindly by two individuals. Statistical analysis was performed using two-tailed, paired Student's *t*-Test. *p*-values < 0.05 were considered significant.

### Cell-cycle assay

The drug treatment was carried out as previously described with either 100 nM of NVP-BGJ398 or 0.01% DMSO. 72 h post drug treatment, cells were trypsinized and fixed with 80% cold ethanol. After PBS wash, cells were stained for 20 min with PE-conjugated anti-Ki67 (BD Biosciences, New Jersey, United States) or the corresponding isotype control (BD Biosciences, New Jersey, United States) at recommended dilutions, followed by PBS wash and Hoechst 33342 (BD Biosciences, New Jersey, United States) staining at a concentration of 2 μg/ml for 15 minutes. Staining was measured using LSR II Flow Cytometer (BD Biosciences, New Jersey, United States). For Hoechst, 355 nm UV laser with 540/50 BP filter and for PE-conjugated Ki67, 488 nm blue laser with 585/42 BP and 550 LP filters were used. The results were analyzed with FlowJo, version 7.6.5 (FlowJo, Oregon, United States). After cell doublet discrimination, the Ki67 negative cell population was gated as G_0_. The isotype control was used to confirm the gating strategy with Ki67. Cell cycle analysis was done using the built in software Cell Cycle Analysis module. Statistical analysis was performed using two-tailed, unpaired Student's *t*-Test. *p*-values < 0.05 were considered significant.

### Viability assay for concentration-response curve and IC_50_ calculations

Cell viability was measured using the CellTiter-Glo Luminescent Cell Viability Assay (Promega, Wisconsin, United States). For each experiment an equal number of cells has been seeded (2.5 × 10^3^ cells per well) onto a non-transparent 96-well plate. The drug treatment was initiated 16 h after seeding and was applied at concentrations ranging from 5 nM to 500 nM. After 0, 24, 48, 72, 96 and 120 h, ATP levels were used as a measure of viability. Relative IC_50_ for 120 h was calculated, both using a non-computational mathematical method based on principle of a right-angled triangle [50] and a Four-Parameter Logistic Function [51] based on a non-linear regression analysis using SigmaPlot (Systat Software, Illinois, United States) version 12.5.0.38.

## SUPPLEMENTARY MATERIALS









## Footnotes

1 www.worldsarcomanetwork.com
