# Supplementary material for: Preclinical evaluation of potential therapeutic targets in dedifferentiated liposarcoma
Source: Oncotarget. 2016 Jul 9;7(34):54583–95. doi: 10.18632/oncotarget.10518 (PMC5342366; doi:10.18632/oncotarget.10518)
Supplement: Supplementary file 1 [file oncotarget-07-54583-s001.pdf]

Preclinical evaluation of potential therapeutic targets in dedifferentiated liposarcoma

Supplementary Materials

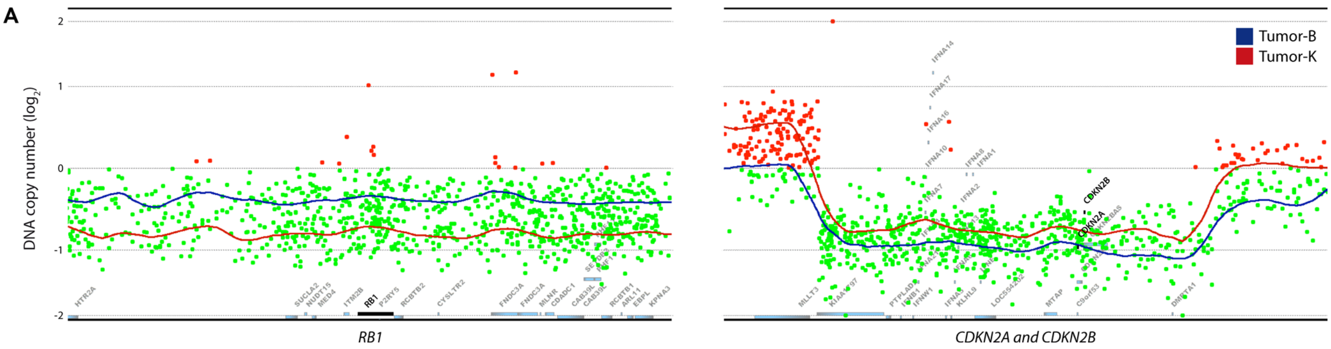

Supplementary Figure S1: DNA copy number changes for tumor-B (blue) and -K (red). (**A**) Detailed view on the genomic region for *RB1*, *CDKN2A* and *CDKN2B*; red dots with a $\log_2$ ratio > 0 and green dots with $\log_2$ ratio < 0. The $\log_2$ ratio is plotted according to position with triangular smoothing of 200 kb.

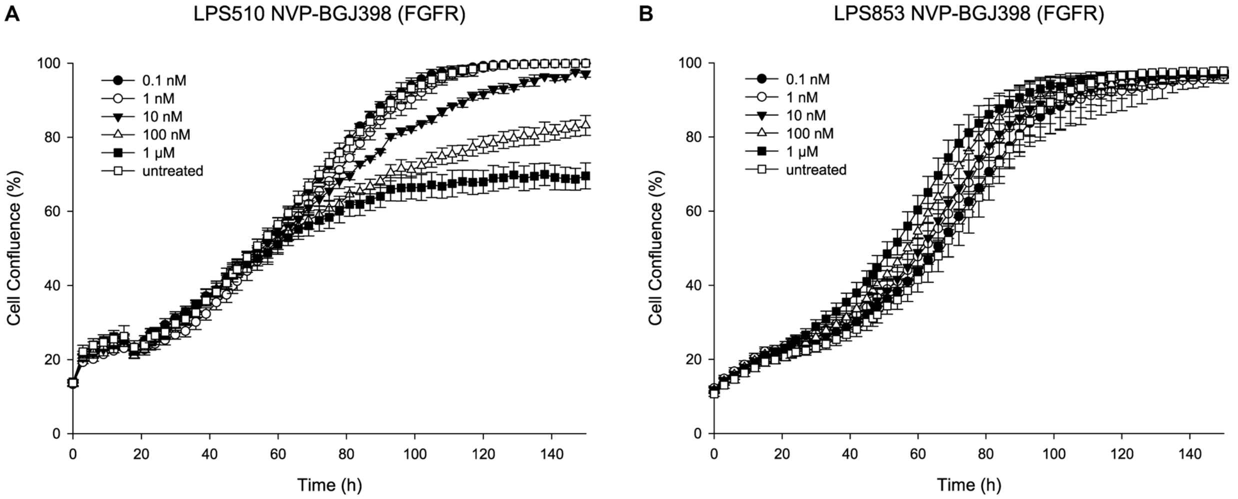

Supplementary Figure S2: The response of two additional DDLPS cell lines to FGFR inhibition with NVP-BGJ398. (**A**) Proliferation of LPS510 cells after inhibition of FGFR with NVP-BGJ398; (n = 1). (**B**) Proliferation of LPS853 cells after inhibition of FGFR with NVP-BGJ398; (n = 1). (A–B) Proliferation measured based on confluence over time; error bars represent standard error (SE) of the measurements. Untreated with DMSO concentration corresponding to that of the highest drug concentration.

Supplementary Table S1: Single nucleotide variations (SNVs) in tumor B, D and K. See Supplementary_Table_S1

Supplementary Table S2: Shared mutations (missense, silent and splice site) between tumor B, D and K

Gene Symbol	Chromosome	Start position	Reference Allele	Mutated Allele	Variant Classification	Mutant Expression
FAM131C	1	16385007	C	T	Silent	No
DOCK7	1	63099204	G	A	Missense Mutation	Yes
TSN	2	122522834	A	G	Missense Mutation	Yes
CYTIP	2	158300390	G	A	Missense Mutation	No
EDEM1	3	5255002	A	G	Splice Site	Yes
MUC12	7	100638279	T	C	Missense Mutation	No
MGAM	7	141727465	G	A	Missense Mutation	No
AQP7	9	33395108	G	A	Silent	No
SVIL	10	29762864	C	T	Missense Mutation	Yes
NDUFA9	12	4778952	G	A	Missense Mutation	Yes
DDX11	12	31242911	C	T	Silent	Yes
COL2A1	12	48372514	C	T	Missense Mutation	No
PPFIA2	12	81693145	C	T	Missense Mutation	No
LTA4H	12	96412657	C	T	Missense Mutation	No
LTA4H	12	96412678	C	T	Missense Mutation	No
WDR66	12	122439498	G	A	Missense Mutation	No
TPP2	13	103301494	G	C	Missense Mutation	Yes
KCNK10	14	88658663	A	G	Missense Mutation	No
DNAAF1	16	84182654	C	T	Missense Mutation	No
COG1	17	71193530	C	G	Missense Mutation	Yes
ASB9	X	15268665	G	A	Missense Mutation	No
MCTS1	X	119740017	G	C	Missense Mutation	No

Supplementary Table S3: DNA copy number differences between tumor B and K. See Supplementary_Table_S3

Supplementary Table S4: DNA copy number and gene expression between tumor B, NRH-LS1 and iMSC#3. See Supplementary_Table_S4
